# A Study of Vicon System Positioning Performance

**DOI:** 10.3390/s17071591

**Published:** 2017-07-07

**Authors:** Pierre Merriaux, Yohan Dupuis, Rémi Boutteau, Pascal Vasseur, Xavier Savatier

**Affiliations:** 1 Normandie University, UNIROUEN, ESIGELEC, IRSEEM, 76000 Rouen, France; remi.boutteau@esigelec.fr (R.B.); xavier.savatier@esigelec.fr (X.S.); 2 Department of Multimodal Transportation Infrastructure, Cerema, 76120 Le Grand Quevilly, France; 3 Normandie University, UNIROUEN, UNIHAVRE, INSA Rouen, LITIS, 76000 Rouen, France; pascal.vasseur@univ-rouen.fr

**Keywords:** motion capture, positioning, evaluation, human motion, robotics

## Abstract

Motion capture setups are used in numerous fields. Studies based on motion capture data can be found in biomechanical, sport or animal science. Clinical science studies include gait analysis as well as balance, posture and motor control. Robotic applications encompass object tracking. Today’s life applications includes entertainment or augmented reality. Still, few studies investigate the positioning performance of motion capture setups. In this paper, we study the positioning performance of one player in the optoelectronic motion capture based on markers: Vicon system. Our protocol includes evaluations of static and dynamic performances. Mean error as well as positioning variabilities are studied with calibrated ground truth setups that are not based on other motion capture modalities. We introduce a new setup that enables directly estimating the absolute positioning accuracy for dynamic experiments contrary to state-of-the art works that rely on inter-marker distances. The system performs well on static experiments with a mean absolute error of 0.15 mm and a variability lower than 0.025 mm. Our dynamic experiments were carried out at speeds found in real applications. Our work suggests that the system error is less than 2 mm. We also found that marker size and Vicon sampling rate must be carefully chosen with respect to the speed encountered in the application in order to reach optimal positioning performance that can go to 0.3 mm for our dynamic study.

## 1. Introduction

Initially developed for gait analysis [1], robotics applications such as UAVs (Unmanned Aerial Vehicle) extensively use Vicon (Oxford, UK) systems. Worldwide numerous Vicon equipped arenas exist such as the arena described in [2]. Studies on quadrotor UAVs [3,4,5,6] use Vicon equipped arenas for purposes such as ground truth for positioning, 3D reconstruction or real-time control given the position estimated by the motion capture system. Marker positions are available at frequencies larger than hundred hertz.

Vicon is one of the key players in optoelectronic motion capture systems based on markers. The trademark is often used as a proprietary eponym for optoelectronic motion capture systems. Other manufacturers such as MotionAnalysis (Santa Rosa, CA, USA), Optitrack (Corvallis, OR, USA) or Qualisys (Göteborg, Sweden) also exist.

Still, few studies investigate the positioning performance of Vicon systems.

Barrows [7] used Vicon MX-F40 cameras while studying aeroelastic resonance in wind tunnels. Markers were placed on a 58 cm long rail. Using the rail, the researchers were able to precisely move the markers. A positioning error slightly larger than 1 mm is found. It corresponds to the error magnitude usually considered as a standard for this type of systems.

Yang et al. [8] have evaluated the positioning performance in 3D with a numerical control drilling machine.The expected positioning precision is given as 20 µm. Different samples are taken in a space corresponding to 400×300×300 ${\mathrm{mm}}^{3}$. Four marker sizes were investigated. They conclude that the marker size does not impact the positioning performance.The main difference is explained by the Vicon camera sensor resolution. The performance is evaluated from 20 positions. Positioning data is collected for 1 s at each location with a motion capture device running at 100 Hz. The metric used is the mean absolute error over all samples. The position-wise performance is not investigated.

Manecy et al. [2] studied the 3D positioning performance in a Vicon equipped arena used for UAVs. The arena is equipped with 17 cameras and represents a space of 6×6×8 $m^{3}$. They did not exactly investigate the Vicon accuracy as no other ground truth setup was used. Markers were manually placed in the arena. The performance metric actually corresponds to the positioning measurement variability. This work demonstrates that the positioning variability is less than 1.5 mm.

Chiari et al. [9] proposes a survey of gait analysis instrumental errors. Their survey shows that inter-distance measurements between markers have extensively been used as the accuracy and precision measurements. Vicon dynamic performance evaluation is based on the ability to perform inter-distance measurements from several kinds of trajectories. They can be grouped into four categories as follows: pendulum test, full volume test, walking test and MAL (Movement Analysis Laboratory, introduced by [10]) test. There is no positioning ground truth system in these cited works.

Diaz et al. [11] proposed evaluating the positioning performance of dynamic objects. This work targets biomedical applications. Several markers were placed on a frame. An electric motor was used to rotate the frame. Several experiment variants were introduced by using two distances between markers, applying two rotational speeds and three motion capture setups: Vicon Mcam-60, Vicon T160 and Hu-m-an Canon Zr300. The inter-marker distance variability was used to assess the performance of the motion capture devices as no positioning ground truth is used. The maximum speed is 0.4 m· $s^{™1}$. The conclusion indicates that, the closer the cameras are to the tracked object, the better the motion capture setup performs. Moreover, better performances are achieved for slow motion patterns on the Hu-m-an Canon Zr300.

To sum up (Table 1), the current state-of-the art papers include either static or low speed experiments (<1 m/s). Contrary to state-of-the art papers, we do not use inter-distance but propose a new framework that relies on marker positioning errors to evaluate the optoelectronic motion capture devices. Marker-wise accuracies and precisions are not investigated in the literature (both robotic and bio mechanical sciences). Volumes found in the Vicon evaluation are rather small compared to environment found in the robotics applications especially for dynamic experiments.

The contributions of this work can be divided into two parts. First, we study the performance of a Vicon motion capture device in a framework corresponding to mobile robotic environment contrary to the state-of-the art papers. The environment encompasses a space large enough to be used in mobile robots applications (UAVs and UGVs). Object motions are close to mobile robotic applications. The system accuracy and precision are assessed for one marker at a time. Secondly, experiments are conducted with both static and dynamic objects with a ground truth positioning setup that does not use motion capture. The static studies was performed with a robot operating 3D motions.

## 2. Experimetal Setup and Evaluation Scheme

The Vicon performance was evaluated with three scenarios. First, we assessed the measurements repeatability also known as the precision of the system. Secondly, a numerically controlled setup was used to estimate the static measure accuracy. Finally, a setup mimicking a blade rotated by an electric motor was used to quantify the dynamic behavior of a motion capture system.

### 2.1. Static Measurement Precision

**Figure 1:**
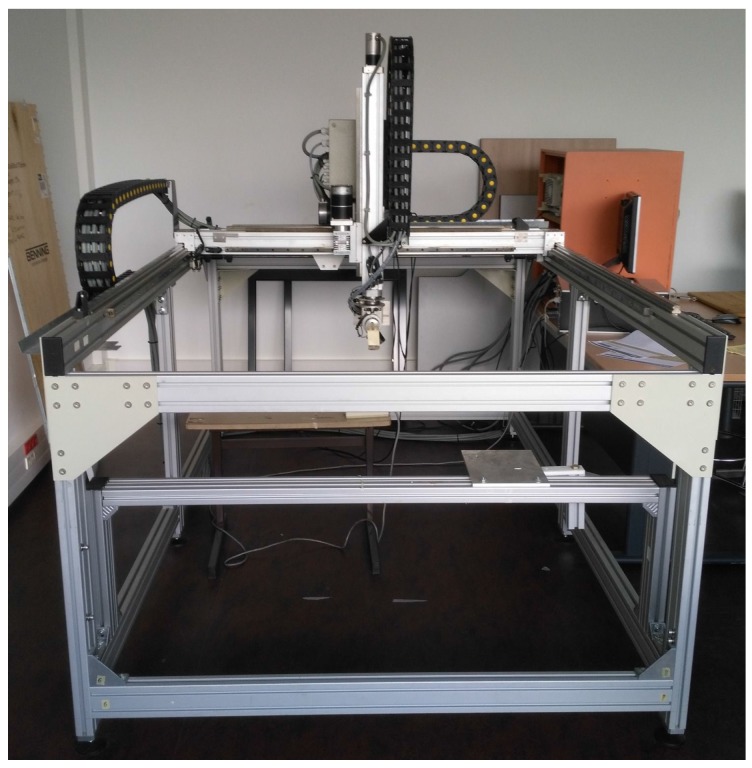
Near field robot used in our static experiments. The probe can move within 2×1.5×1 $m^{3}$, 10 µm positioning sensitivity.

The Electromagnetic compatibility (EMC) laboratory from IRSEEM (Embedded Electronic Systems Research Institute) has a numeric controlled 4-axis robot used to perform EMC near field experiments (Figure 1). Its positioning specifications allow to reach a position with a 10 μm resolution. The probe can be moved in a space of 2×1.5×1 $m^{3}$. The robot was used to perform repeatability measurement evaluation and static measurement performance quantification.

A Vicon marker was placed on the probe monitored by eight T40S cameras. Marker positions are recorded in the robot and Vicon reference frames. Both frames are aligned as follows.

A list P of at least eight 3D points $p_{i}$ are used. Each point $p={\left[x,y,z,1\right]}^{T}$ in the Vicon frame and its counterpart in the robot frame $p_{r}$ are related by a rigid transform as both frames use a calibrated metric representation:

(1) $$p=\left[\begin{array}{cc} R & t\\ 0^{T} & 1 \end{array}\right]\cdot p_{r},$$

(2) $$p=H\cdot p_{r},$$

with:
R:a 3×3 rotation matrix with detR=1 and $R^{T}=R^{™1}$,t:a 3×1 translation vector,0:a 3×1 zero vector.

Estimating the rigid transform between both frames can be expressed as a least-square problem as follows:

(3) $$p=H\cdot p_{r},$$

(4) $$p=\left[\begin{array}{c} h_{1}\\ h_{2}\\ h_{3}\\ h_{4} \end{array}\right]\cdot p_{r}=\left[\begin{array}{cccc} h_{1,1} & h_{1,2} & h_{1,3} & h_{1,4}\\ h_{2,1} & h_{2,2} & h_{2,3} & h_{2,4}\\ h_{3,1} & h_{3,2} & h_{3,3} & h_{3,4}\\ h_{4,1} & h_{4,2} & h_{4,3} & h_{4,4} \end{array}\right]\cdot p_{r},$$

(5) $$p=\left[\begin{array}{cccc} p_{r}^{T} & 0 & 0 & 0\\ 0 & p_{r}^{T} & 0 & 0\\ 0 & 0 & p_{r}^{T} & 0\\ 0 & 0 & 0 & p_{r}^{T} \end{array}\right]\cdot \left[\begin{array}{c} h_{1}\\ h_{2}\\ h_{3}\\ h_{4} \end{array}\right],$$

(6) $$\left[\begin{array}{c} x\\ y\\ z\\ 1 \end{array}\right]=\left[\begin{array}{cccc} p_{r}^{T} & 0 & 0 & 0\\ 0 & p_{r}^{T} & 0 & 0\\ 0 & 0 & p_{r}^{T} & 0\\ 0 & 0 & 0 & p_{r}^{T} \end{array}\right]\cdot \left[\begin{array}{c} h_{1}\\ h_{2}\\ h_{3}\\ h_{4} \end{array}\right],$$

with:
R =$H_{1:3,1:3},$t =$H_{1:3,4},$0 =a 4×1 zero vector.

Equation (6) can be written in the form A·x=b with:
*n*:the number of points used in the rigid transform estimation,A:a 4n×16 matrix,b:a 4n×1 vector,x:a 16×1 vector including the rigid transform parameters.

(7) $$\overset{A}{\overset{︷}{\left[\begin{array}{cccccccccccccccc} x_{M}^{1} & y_{M}^{1} & z_{M}^{1} & 1 & 0 & \cdots & 0\\ 0 & 0 & 0 & 0 & x_{M}^{1} & y_{M}^{1} & z_{M}^{1} & 1 & 0 & \cdots & 0\\ 0 & \cdots & 0 & x_{M}^{1} & y_{M}^{1} & z_{M}^{1} & 1 & 0 & \cdots & 0\\ 0 & \cdots & 0 & x_{M}^{1} & y_{M}^{1} & z_{M}^{1} & 1\\ \vdots & \vdots & \vdots & \vdots & \vdots & \vdots & \vdots & \vdots & \vdots & \vdots & \vdots & \vdots & \vdots & \vdots & \vdots & \vdots \\ x_{M}^{n} & y_{M}^{n} & z_{M}^{n} & 1 & 0 & \cdots & 0\\ 0 & 0 & 0 & 0 & x_{M}^{n} & y_{M}^{n} & z_{M}^{n} & 1 & 0 & \cdots & 0\\ 0 & \cdots & 0 & x_{M}^{n} & y_{M}^{n} & z_{M}^{n} & 1 & 0 & \cdots & 0\\ 0 & \cdots & 0 & x_{M}^{n} & y_{M}^{n} & z_{M}^{n} & 1 \end{array}\right]}}\cdot \overset{x}{\overset{︷}{\left[\begin{array}{c} h11\\ h12\\ h13\\ h14\\ h21\\ h22\\ h23\\ h24\\ h31\\ h32\\ h33\\ h34\\ h41\\ h42\\ h43\\ h44 \end{array}\right]}}=\overset{b}{\overset{︷}{\left[\begin{array}{c} x_{V}^{1}\\ y_{V}^{1}\\ z_{V}^{1}\\ 1\\ x_{V}^{2}\\ y_{V}^{2}\\ z_{V}^{2}\\ 1\\ \vdots \\ x_{V}^{n}\\ y_{V}^{n}\\ z_{V}^{n}\\ 1 \end{array}\right]}}$$

with:
$x_{M}^{1}$:is *x* coordinate of point $p^{1}$ in the world frame,$x_{V}^{1}$:is *x* coordinate of point $p^{1}$ in the Vicon frame.

In order to reduce invertability problems, $A^{t}A\cdot x=A^{t}b$ is solved. Once x is found, the rotation matrix R is forced using the SVD operation:

(8) $$\left[\begin{array}{c} U,S,V \end{array}\right]=svd\left(R\right),$$

(9) $$R_{\mathrm{ortho}}=V\cdot U^{t}.$$

The rotation matrix and translation between the Vicon and robot frame are estimated. It can be used to measure the Vicon measurement repeatability and accuracy in a static framework.

The Euclidean distance is used to quantify the Vicon performance.

### 2.2. Dynamic Measurement Evaluation

Contrary to the state-of-the art, dynamic measurements were performed at speeds encountered in mobile robotic environments. We were aiming at finding a ground truth setup with a sampling rate higher than the Vicon sampling rate used in our experiments (100 Hz).

**Figure 2:**
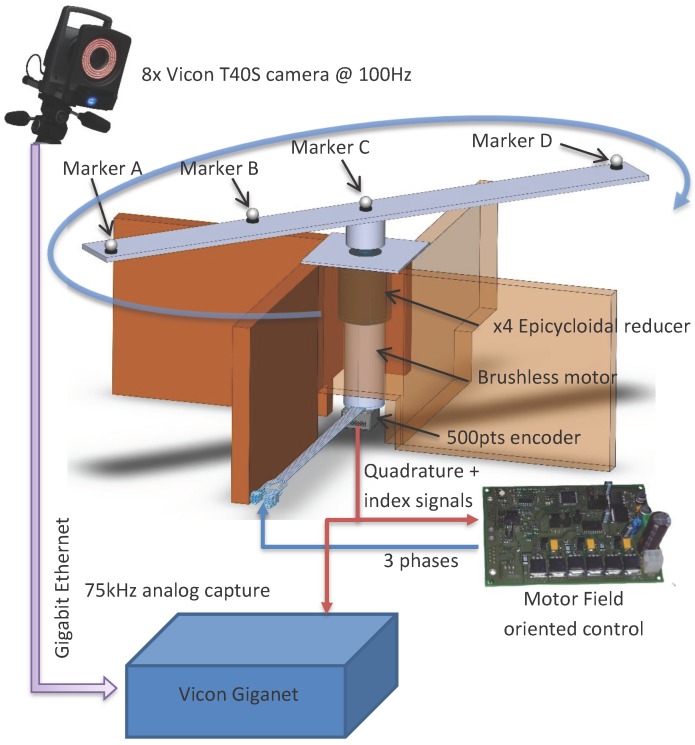
Rotor used in the dynamic experiments.

We created a setup able to reach high speed made of a blade and a rotor. As it can be seen in Figure 2, the system is made of an electric motor, a gear box and a blade where four markers are sticked. Mechanical slacks are controlled in order to reduce their impact on the ground truth precision. The engine torque is controlled with a vector control command in order to ensure torque stability. The ground truth position is expressed as the angular position about the rotor axis. The angular ground truth is obtained from a 500 pulses per revolution quadrature encoder installed on the motor axle. Synchronisation with the motion capture system is ensured by plugging the resulting encoder signals into the Vicon Giganet. Encoder signals are sampled at 75 kHz.

A 0.045° angular resolution is achieved thanks to a gear reduction ratio of 4 and by exploiting rising and falling edges. Markers A and D are placed at each end of the blade 30 cm away from the motor axis. It results in a positioning resolution of 0.235 mm. The Vicon system accuracy is expected to be about one millimeter given reference [7]. The actual positioning resolution might be too large. As a result, we interpolated the angular position up to the encoder sampling rate, i.e., 75 kHz.

The resulting angular resolution now depends on the motor spinning speed. The expected accuracy for markers A and D lies between 10 µm and 102 µm for speeds of 100 rpm and 1000 rpm, respectively. These values are more suitable to evaluate a system with an expected accuracy of 1 mm.

As mentioned previously, we track and evaluate the estimated angular position of each marker and compare them to the ground truth angular position. As a result, the transformation T between the Vicon frame and the angular frame must be estimated with T defined as a similarity :

(10) $$T=\left[\begin{array}{cc} sR & t\\ 0^{T} & 1 \end{array}\right].$$

In order to avoid numerical instabilities or realignment issues, we used two approaches to project the measurements performed in the Vicon frame into the ground truth frame, i.e., angular positions. Contrary to static study, the marker location on the blade is considered as unknown, so we will estimate it (*s* scale parameter).

The blade initial position is unknown with respect to the world frame. Moreover, the marker locations can be roughly measured on the blade, but their position might not be accurate enough to ensure a proper transformation estimation.

In order to estimate the transformation between the Vicon frame and the angular frame, we must transform the angular coordinates to 3D points as follows :

(11) $$x_{t}={\left[\cos\theta_{t}\;\sin\theta_{t}\;0\right]}^{T},$$

with:
$\theta_{0}$$=0^{\circ }$,$\theta_{t+1}$$=\theta_{t}+0.045^{\circ }$.

Consequently, $x_{t}$ will be moving on a unit circle encompassed in the x™y plane.

$x_{t}^{\prime }$, the counterpart of $x_{t}$ in the Vicon frame, is related by the following equation :

(12) $$x_{t}^{\prime }=T\cdot x_{t}.$$

As corresponding points are known from the sensor synchronization, we employed :

- Uyemana’s method [12] to estimate *s*, R and t. Let us define $X=\left\{x_{0},x_{1},\cdots ,x_{n™1},x_{n}\right\}$ and $X^{\prime }=\left\{x_{0}^{\prime },x_{1}^{\prime },\cdots ,x_{n-1}^{\prime },x_{n}^{\prime }\right\}$, the corresponding sets of three-dimensional points. Uyemana’s solution aims at minimizing :
  (13) $$e=\frac{1}{n}\sum_{i=1}^{n}{∥x_{i}^{\prime }™\left(sRx_{i}+t\right)∥}^{2}.$$
  It is performed by first computing the X and $X^{\prime }$ means :
  (14) $$\mu_{x}=\frac{1}{n}\sum_{i=1}^{n}x_{i},$$
  (15) $$\mu_{x}^{\prime }=\frac{1}{n}\sum_{i=1}^{n}x_{i}^{\prime }.$$
  Secondly, the covariance matrix of X and $X^{\prime }$ is computed :
  (16) $$\Sigma_{{\mathrm{xx}}^{\prime }}=\frac{1}{n}\sum_{i=1}^{n}\left(x_{i}^{\prime }™\mu_{x}^{\prime }\right){\left(x_{i}™\mu_{x}\right)}^{T}.$$
  Thirdly, a singular value decomposition is performed on $\Sigma_{{\mathrm{xx}}^{\prime }}$ :
  (17) $$USV^{T}=SVD\left(\Sigma_{{\mathrm{xx}}^{\prime }}\right).$$
  Finally, let us consider the D defined as follows :
  (18) $$D=\left\{\begin{array}{cc} diag(1,1,sign\left(\det\left(\Sigma_{{\mathrm{xx}}^{\prime }}\right)\right)*1), & \mathrm{if}\;rank(\Sigma_{{\mathrm{xx}}^{\prime }})=3,\\ diag(1,1,sign\left(\det\left(U\right)*\det\left(V^{T}\right)\right)*1), & \mathrm{if}\;rank(\Sigma_{{\mathrm{xx}}^{\prime }})=2. \end{array}\right.$$
  *s*, R and t are computed as follows :
  (19) $$R=UDV^{T},$$
  (20) $$s=\frac{1}{\sigma_{x}^{2}}tr\left(SD\right),$$
  (21) $$t=\mu_{x}^{\prime }-sR\mu_{x},$$
  where:
  (22) $$\sigma_{x}^{2}=\frac{1}{n}\sum_{i=1}^{n}∥x_{i}^{\prime }™\mu_{x}∥.$$
- A second approach uses the fact that the markers are placed on a rotating blade. As a consequence, their trajectories are expected to be performed on a 3D plane.
  The 3D plane equation can be estimated as follows. A point $p={\left[x\;y\;z\;1\right]}^{T}$ in the Vicon frame lying on the 3D plane $N={\left[a\;b\;c\;d\right]}^{T}$ must fulfill the condition $p^{t}\cdot N=0$. From at least four points, N can be estimated. N is the null space of P with $P={\left[p_{1}^{t},p_{2}^{t},p_{3}^{t},p_{4}^{t}\right]}^{T}$. In practice, more than four points are used. As $null\left(P^{t}\cdot P\right)=null\left(P\right)$, the following operations are performed :
  (23) $$\left[\begin{array}{c} U,S,V \end{array}\right]=svd\left(P^{t}\cdot P\right).$$
  $N=v_{4}$ with $V=\left[\begin{array}{c} v_{1},v_{2},v_{3},v_{4} \end{array}\right].$
  Each point is consequently transformed in a new frame that aligns the *z*-axis with N. The final step requires to fit a circle to the resulting point cloud. The circle radius corresponds to *s* and the circle center to t.
  One last variable must be estimated: the constant angular offset that exists between point extracted from the Vicon and the encoder location. In fact, as the angular position of the blade is not known at the first timestamp due to the fact that the encoder is incremental. Moreover, the reduction gear ratio brings a ground truth uncertainty depending on the rotation speed. As a result, recordings are performed once the rotation speed is stable. It also allows to obtain a unique speed for each dataset. We find the angular offset by incrementally testing offset values β that correspond to a multiple of the resulting angular resolution. β is set as :
  (24) $$min_{\beta }\sum {\left(\theta_{i}™\alpha_{i}+\beta \right)}^{2},$$
  with:
θ:the groundtruth angle at timestamp i,α:the Vicon angle at timestamp i,β:the offset angle to be estimated.
  Once β is found, N and angle β are used in axis-angle rotation conversion to a rotation matrix R.

The Euclidean distance is used as the performance metric of the Vicon positioning for both transformation estimations.

## 3. Results for Static Experiments

**Figure 3:**
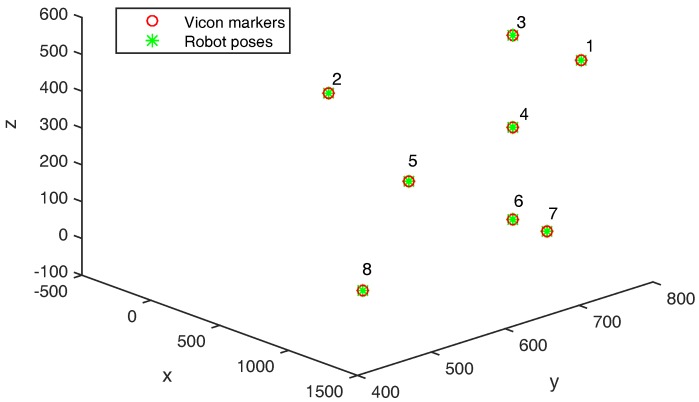
Eight static points after Vicon and robot frame alignment.

**Figure 4:**
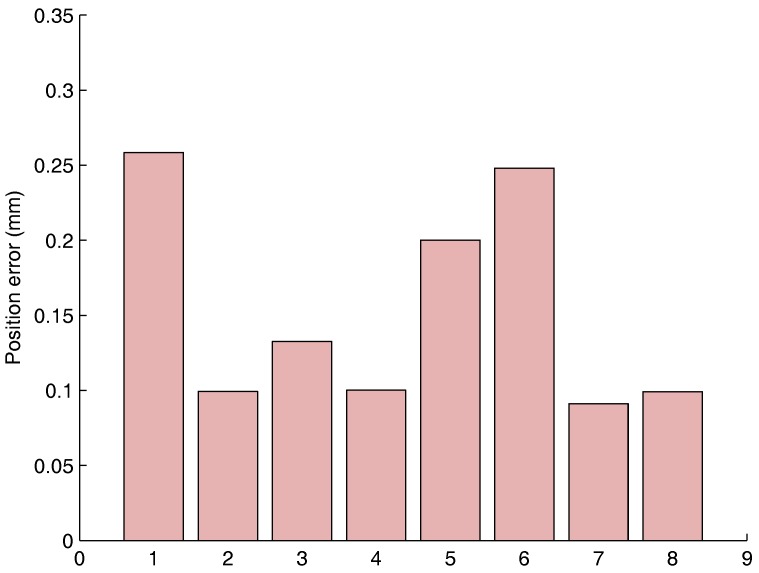
Static point positioning error.

Figure 3 shows the Vicon markers represented in the Robot frame thanks to the transformation H found with the Least-square method explained in Section 2.1. It can be seen that both point clouds perfectly align. The transformation is properly estimated with a mean squared error of 0.224 mm after frame alignment. In Figure 4, it can be seen that the retro-projection error is less than 0.255 mm for all markers. The Mean Average Error (MAE) is 0.153 mm and Root Mean Squared Error (RMSE) is 0.154 mm.

**Figure 5:**
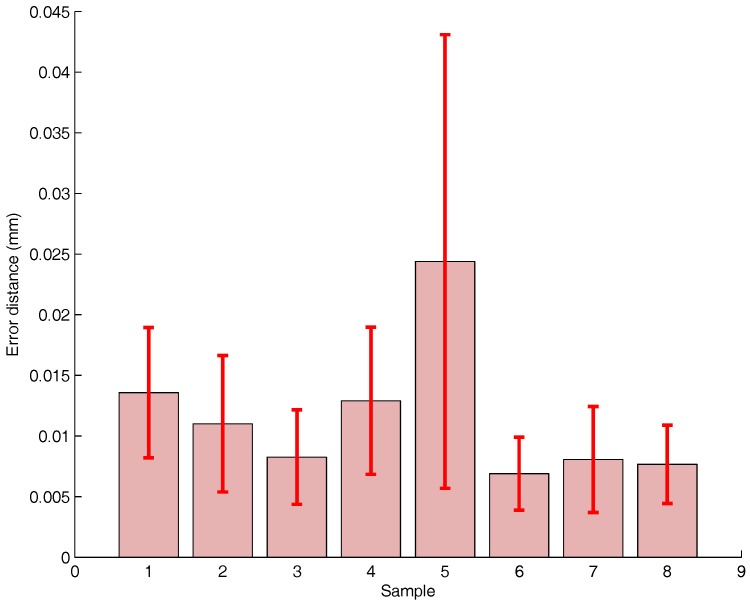
Static point positioning variability. Bar indicate the standard deviation—red whiskers show the variability range.

In Figure 5, we investigate the Vicon accuracy and precision. A marker is placed at a location with the robot arm. Its position is sampled at 100 Hz over 1 s.

For each marker, Figure 5 shows the position accuracy, i.e., the bar graph, as well as the precision, i.e, the whiskers. The Vicon accuracy is really high with a worst accuracy of 25 µm. The precision is closely related to the magnitude of the accuracy. Marker 5 has an accuracy of 25 µm and a precision of 19 µm, while Marker 6 has an accuracy of 7 µm and a precision of 4.5 µm. It results in a coefficient of variation equal to 0.76 and 0.64, respectively. It could be regarded as quiet high. Still, the accuracy is so high that the coefficient of variation does not have any significant impact.

The precision found in our study is better than [2]. Moreover, our marker-wise study shows relatively significant difference in accuracies with values spanning from 7 µm to 25 µm. Still, those values are not significant for robotic applications.

## 4. Results for Dynamic Experiments

### 4.1. Extrinsic Calibration Method Comparison

The blade setup was used to generate several datasets as it can be seen in Table 2. Two markers were placed at each end of the blade. A third marker was placed between the blade rotation axis and the final marker.

**Figure 6:**
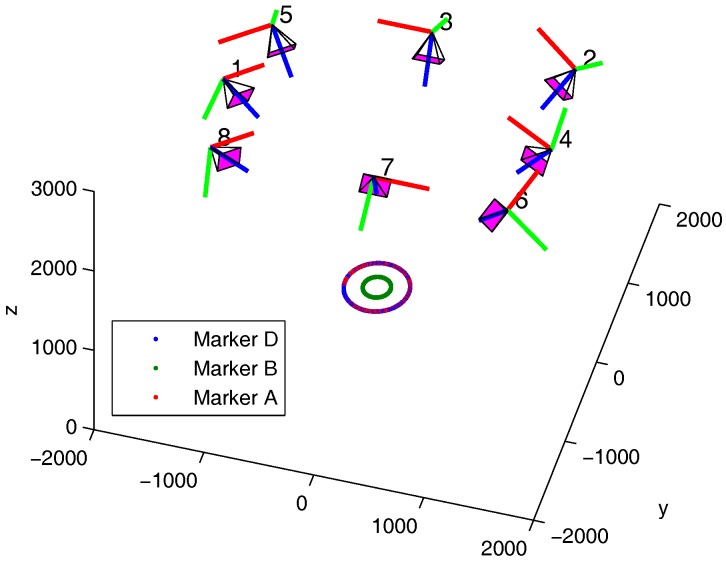
Trajectories of markers A, B and D. Camera locations and orientations obtained from the Vicon calibration wand procedure.

As it can be seen in Figure 6, eight Vicon cameras were placed around the blade in a convenient fashion. The camera poses are represented in 3D. The circles correspond to the marker trajectories.

Eight datasets were recorded. They encompass different rotation speeds as well as Vicon sampling speeds. Vicon sampling speeds investigated are equal to 100 Hz and 200 Hz. For all experiments, the coder sampling rate was set to 75 kHz. Dataset 8 includes only one marker. In fact, markers A and D flew away due to the high rotational speeds.

First, we investigate the frame alignment performance. Two methods were investigated (c.f. Section 2.2): Uyemana method and an approach that uses a plane estimation followed by a circle fitting that we named “Plane+Circle”. As it can be seen in Table 3, the resulting rotations are close. The marker positions on the blade are also really close with no difference between the two models (c.f. Table 4). From Table 5, it can be seen that the model errors are close despite slight differences in the translation estimation (c.f. Table 6).

**Figure 7:**
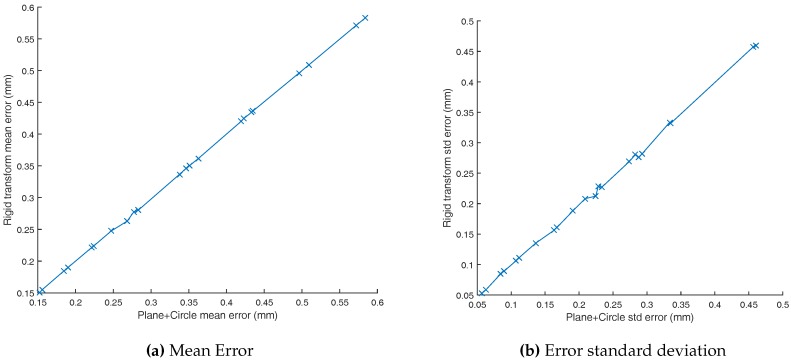
Comparison of both extrinsic calibration models. The mean difference between both models is equal to 2.23%.

In Figure 7, we compare the average error achieved by both calibration datasets. Each point corresponds to the mean positioning error of a given marker taken from an experiment. As it can be seen, both model mean error and standard deviation are linearly correlated. Both models lead to sub millimeter mean error on all datasets. The mean error spans from 0.15 mm to 0.58 mm for both models. The standard deviation ranges from 0.05 mm to 0.46 mm. Overall, the coefficient of variation varies from 0.33 to 0.79. The standard deviation is correlated with the mean error as smaller coefficients of variation are reached for small mean errors and larger coefficients for larger mean errors.

The models are really close to each other. As a result, for the rest of this paper, we report “Plane + Circle” positioning results.

### 4.2. In-Plane versus Out-of-Plane Errors

**Figure 8:**
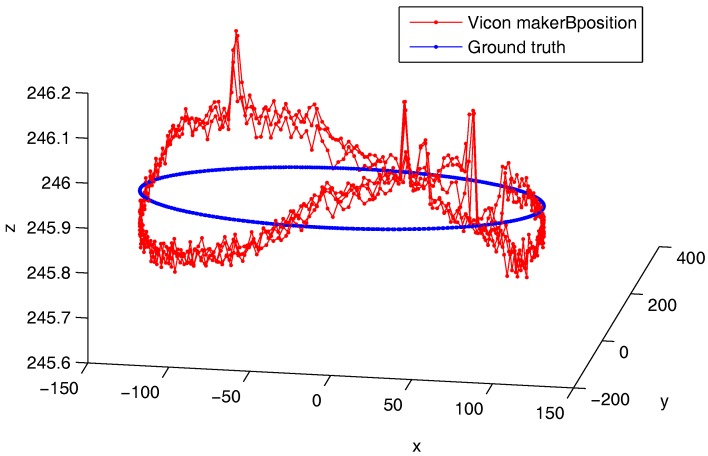
Marker B trajectory from dataset 1 and ground truth (axis in millimeters).

Figure 8 shows the ground truth trajectory and the Vicon-based trajectory from marker B in dataset 1 resulting in its lowest speed. As it can be seen, there is an oscillation about the xy plane. As it can be noticed, the scale of the *z*-axis is zoomed in compared to the other two dimensions. However, we can suspect a measurement error or mechanical problems such as blades oscillations, motor carrying structure displacement, etc.

**Figure 9:**
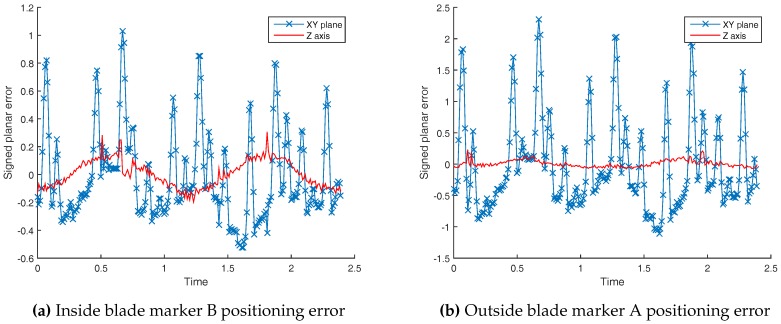
In-plane and Out-of-plane positioning error from Dataset 1.

**Figure 10:**
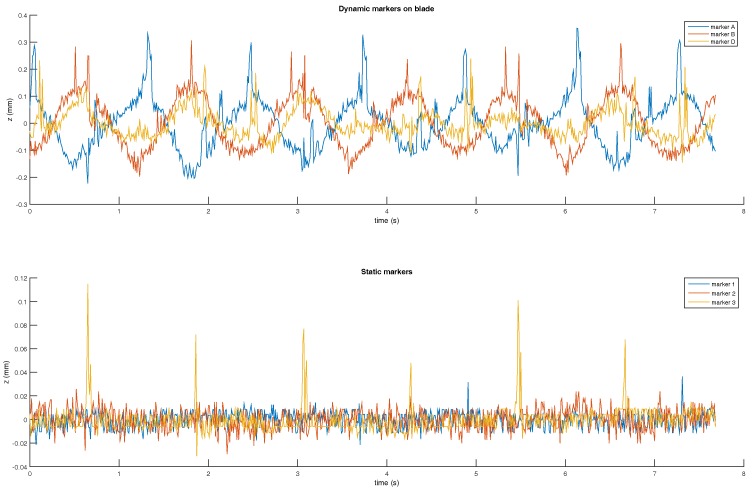
Out-of-plane error over time.

To check a blade flexural oscillation problem, we have plotted the *z*-axis error of outside marker A and inside marker B in Figure 9a,b. The vertical axis of the plots are not at the same scale. Still, the Z error amplitude are close to each other, and similar to Figure 10. The blade was made of 4 mm thick aluminum 2017A. Due to the blade small speed and its mechanical robustness, it is less likely that we are observing a blade mechanical oscillation.

To check the last hypothesis, we have placed three Vicon markers on the static structure (Figure 2, the marker are placed on the top of the brown panels). Figure 10 represents the Z displacements of fix and dynamic makers. We can notice that the vertical motions of the static makers are rather small compared to dynamic markers. The fix marker motions are within the Vicon measurement noise, and the values are closed to the repeatability error found in Figure 5. Consequently, ground truth static structure displacement can be discarded.

**Figure 11:**
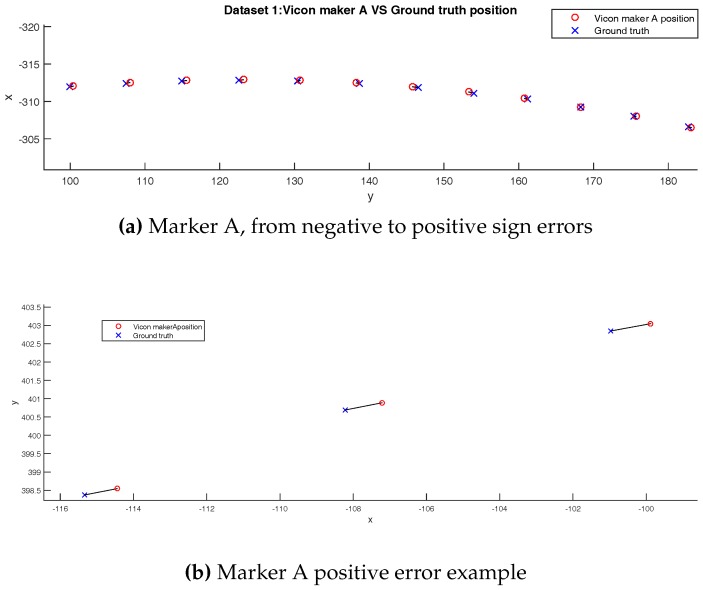
Planar errors, Dataset 1 (axis in millimeters).

Nevertheless, in Figure 9, the *z*-axis error is small compare to xy planar error. To confirm this fact, we plot an xy view of marker A ground truth and measured position. Black lines between ground truth and measured positions represent the distances shown as blue lines in Figure 11.

**Figure 12:**
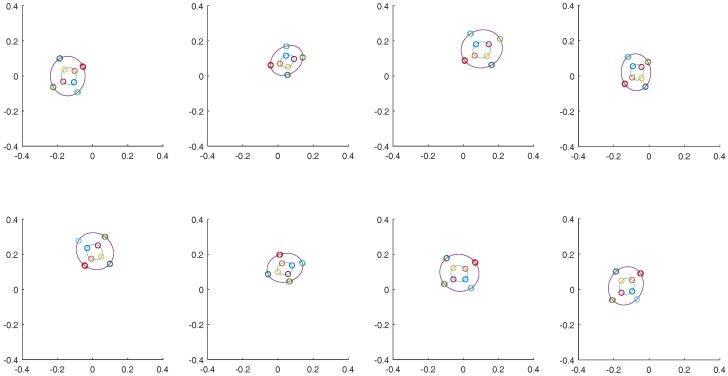
Marker trajectories in the eight Vicon camera unit planes—circles on ellipses indicate locations of main outliers of the Figure 9—A color represents the same marker seen from different cameras. Marker B trajectory corresponds to the small ellipse. Marker A trajectory corresponds to the large ellipse.

All things considered, errors observed are likely due to measure process rather than mechanical uncertainties. The Vicon system is based on multi-views marker triangulation. These kinds of methods could be influenced by the camera location, distribution as well as calibration. To check that we did not evaluate the system in degraded case, we have plotted the marker trajectories in the unit plane of each Vicon camera (c.f. Figure 12). The markers’ retro-projection uses the camera positioning shown in Figure 6. Figure 12 shows that the maker trajectories are mostly centered in the image given the lens used in our experimentations.

After investigating extrinsic calibration and ground performance, our comparison process could be considered as correct. We now focus on markers’ speed influence on the measurement error and final result investigation.

### 4.3. Error versus Speed

**Figure 13:**
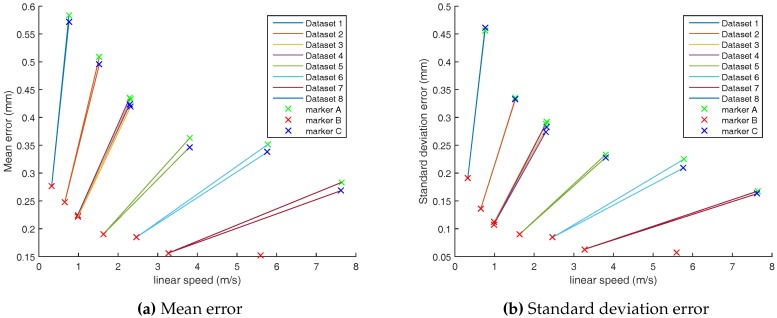
Error vs. linear speed for each dataset.

We have experimented different speeds in eight datasets (c.f. Table 2). We explore the accuracy with respect to linear speeds. Results are shown in Figure 13a,b.

Firstly, the accuracy is generally better as the speed increases. It seems to be inversely proportional to the speed. The mean errors and their standard deviations follow the same trend.

Secondly, with the motion chosen in experimental setup, i.e., circular motion (Figure 2), the small diameter marker (B) error is fifty percent lower than outside diameter markers (A and D).

We have three hypotheses to explain this phenomenon :

- The camera intrinsic calibration model may be better to correct distortions in the center of the image than on borders. As shown in Figure 12, the B marker is more often close to the image center than markers A and D.
- In our experimentations, we used 12.7 mm markers. For speeds lower than 1.3 m/s, the marker will overlap on two consecutive images at 100 Hz acquisition rate. In Figure 13a,b, the error seems to flatten for speeds larger than 1.3 m/s for dataset 1 to 6. Then, a new stage is reached for datasets 7 and 8, where the Vicon sampling rate was set to 200 Hz. At 200 Hz, the marker overlapping is avoided for speeds larger than 2.6 m/s.
- The fact that the error decreases with the speed could be due to marker tracking algorithms with higher speed measurement uncertainty at low speeds. Datasets recorded in our experiments at 200 Hz have linear speeds larger than this value.

All things considered, the worst mean absolute error (Dataset 1) is lower than 0.5 mm. In the static evaluation (Section 3), we have found the largest error was equal to 0.25 mm with standard deviation of 0.015 mm. This static error value is close to internal marker B.

### 4.4. Final Results

Table 7 shows that the Z error is not significant compared to XY components. This outcome confirms the observations made in Section 4.2.

**Figure 14:**
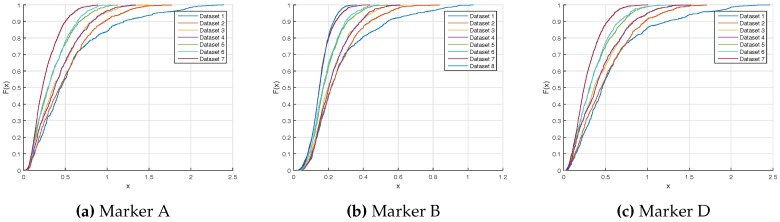
Position error cumulative distribution functions (*x*-axis in millimeters).

Figure 14 presents the cumulative distribution function error of each marker. As it can be seen, the faster speed and the small diameter marker have the lower errors and less variability. Markers A and D have the same shape, due to their opposite location on the rotor. (Figure 2). On the one hand, for the worst case, Dataset 1 and markers A and D, the Vicon measurement is better than 2 mm at 3 σ. On the other hand, the best case, Dataset 8 and marker B, Vicon measurement is better than 0.3 mm at 3 σ.

Table 8 summarized the final results for three linear speed ranges: less than 1 m/s, 1 to 3 m/s and more than 3 m/s. To compute these values, we have averaged all the samples in the same range. We also considered the 1 σ, 2 σ and 3 σ error. It can be seen that sub-millimeter positioning error is reached for speed larger than 3 m/s. For lower speed, the error is still fair with errors lower than 2 mm.

## 5. Conclusions

In this paper, we proposed an optoelectronic motion capture evaluation protocol. It includes a new mechanical setup that encompasses sensors that give ground truth positioning. It allows for leveraging limits found in existing works that are based solely on inter-marker distances studies [9]. We are able to provide marker-wise absolute positioning performance. We studied the device behavior for two distinct scenarios, namely static and dynamic. Speeds and trajectories chosen are compatible with mobile robotic or UAV applications as well as other real life applications. We propose a circular trajectory that, to the best of our knowledge, has never been used to evaluate motion capture systems. Our evaluation was based on an external ground truth not relying on another motion capture device.

Static experiments have shown a mean absolute positioning error of 0.15 mm and a really low variability of 0.015 mm. Consequently, a Vicon system has an excellent precision and high accuracy for static cases.

Dynamic experiments have highlighted interesting aspects. In fact, faster displacements of the marker lead to lower errors. The error can be reduced by 40% for the same object if it moves at higher speeds. This phenomena might be related to tracking error and marker size. In fact, if the marker does not move sufficiently between two samples, the motion observed by the cameras is within the segmentation noise of each camera. Still, our study found that the Vicon positioning error can be considered as lower than 2 mm from low to high speed experiments. Moreover, the positioning variability is better for static experiments.

As a result, the marker size and the Vicon sampling rate should be properly tuned with respect to the speed displacements encountered in the monitoring applications to reach the Vicon optimal performance.

Our future work will be focused on performing other trajectories in order to evaluate the robustness of the positioning in a dynamic environment. First, we will perform linear motion at speeds similar to the circular trajectories found in our work. Secondly, we will use a Kuka robot in order to mimic human being or animal motion that could be considered as more complex motion than pure rotation or translations. Finally, the same setup will be used to mimic UAV acrobatic-like motions that are performed at higher speeds and less constrained than living being motions. 

## Figures and Tables

**Table 1:** Summary of existing Vicon evaluation studies.

Reference	Space	Precision Evaluation	Static	Dynamic
[2]	6×6×8 $m^{3}$	✖		
[7]	4.6×73 $m^{2}$ wind tunnel		0.58 m displacements	
[8]	0.4×0.3×0.3 $m^{3}$	✖		
[11]	2.5×1×1 $m^{3}$			linear motion (maximum speed of 0.6 m/s)
Our work	2×1.5×1 m^3^	✖	✖	External ground truth (max. speed of 7.6 m/s)

**Table 2:** Dataset collected with the rotor setup.

Dataset Number	Motor Speed (rpm)	Blades Speed (rpm)	Usable Markers	Vicon Sample Rate (Hz)	Encoder Sample Rate (kHz)
1	100	25	A B D	100	75
2	201	50.25	A B D	100	75
3	304	76	A B D	100	75
4	301	75.25	A B D	100	75
5	499	124.75	A B D	100	75
6	756	189	A B D	100	75
7	1000	250	A B D	200	75
8	1711	427.75	B	200	75

**Table 3:** Reference frame rotation estimation.

Model	Roll (°)	Pitch (°)	Yaw (°)
Uyemana Method	−1.33	2.38	−65.00
Plane + Circle	1.39	2.58	−64.88
Model distances	2.72	0.20	0.12

**Table 4:** Marker radial position.

Model	Marker A (mm)	Marker B (mm)	Marker D (mm)
Uyemana Method	291.34	124.32	291.18
Plane + Circle	291.34	124.32	291.18
Deviation	0	0	0

**Table 5:** Model errors.

Model	Mean Absolute Error (mm)
Uyemana Method	0.5090
Plane + Circle	0.5093

**Table 6:** Reference frame translation estimation.

Model	Tx (mm)	Ty (mm)	Tz (mm)
Uyemana Method	−31.75	116.98	246.26
Plane + Circle	−28.60	133.19	246.75
Model distances	3.15	16.21	0.49

**Table 7:** Dynamic experimentation results in millimeter. 3D error and in-plane error ($Error_{XY}$). Dataset 8, markers A and B are not workable. Due to high speed, the centrifugal force unstuck markers.

Dataset	Marker	Estimated Radius	Linear Speed (m/s)	Error mean	${\mathrm{Error}}_{\mathrm{XY}}$Mean	Error Std	${\mathrm{Error}}_{\mathrm{XY}}$Std
1	D	291.34	0.76	0.58	0.57	0.46	0.46
1	B	124.32	0.32	0.28	0.25	0.19	0.20
1	A	291.18	0.76	0.57	0.57	0.46	0.46
2	D	291.34	1.53	0.51	0.50	0.33	0.34
2	B	124.32	0.65	0.25	0.22	0.14	0.15
2	A	291.19	1.53	0.50	0.49	0.33	0.34
3	D	291.35	2.32	0.43	0.42	0.29	0.30
3	B	124.33	0.99	0.22	0.19	0.11	0.12
3	A	291.19	2.32	0.42	0.41	0.28	0.29
4	D	291.35	2.29	0.44	0.42	0.29	0.30
4	B	124.33	0.98	0.22	0.19	0.11	0.12
4	A	291.19	2.29	0.42	0.42	0.27	0.28
5	D	291.35	3.81	0.36	0.34	0.23	0.24
5	B	124.35	1.62	0.19	0.16	0.09	0.10
5	A	291.19	3.80	0.35	0.34	0.23	0.23
6	D	291.36	5.77	0.35	0.33	0.22	0.23
6	B	124.38	2.46	0.18	0.15	0.08	0.09
6	A	291.20	5.77	0.34	0.33	0.21	0.21
7	D	291.42	7.63	0.28	0.25	0.17	0.18
7	B	124.62	3.26	0.16	0.13	0.06	0.07
7	A	291.26	7.63	0.27	0.25	0.16	0.17
8	D	180.09	8.07	229.38	229.38	110.89	110.89
8	B	125.06	5.60	0.15	0.13	0.06	0.06
8	A	315.53	14.13	401.63	401.63	193.92	193.92

**Table 8:** Dynamic experimentation synthetic results in millimeters.

Speed Range	Mean Error	Std Error	RMSE	1 σ Error (68.27%)	2 σ Error (95.45%)	3 σ Error (99.73%)
<1 m/s	0.3543	0.2439	0.5236	0.3861	0.9156	1.2073
[1, 3] m/s	0.3863	0.2474	0.4171	0.4745	0.8869	1.1583
>3 m/s	0.2823	0.1682	0.3293	0.3424	0.6165	0.8330
